# Supplementary material for: Fecal microbiota transplant rescues mice from human pathogen mediated sepsis by restoring systemic immunity
Source: Nat Commun. 2020 May 11;11:2354. doi: 10.1038/s41467-020-15545-w (PMC7214422; doi:10.1038/s41467-020-15545-w)
Supplement: Supplementary file 1 — Supplementary Information [file 41467_2020_15545_MOESM1_ESM.docx]

**Fecal microbiota transplant rescues mice from human pathogen mediated sepsis by restoring systemic immunity**

**Kim et al.**

**Supplementary Information:**

Supplementary Table 1-4

Supplementary Figures 1-6

**Supplementary Table 1. Antibiotic susceptibility of microbes isolated from ICU patient stool sample**

| **Cultured microbes** | **Antibiotic resistant profile** |
| --- | --- |
| *Candida albicans* | Amphotericin B^S^, micafungin^S^, flucytosine^S^, voriconazole^S^, itraconazole^S^, fluconazole^S^ |
| *Enterococcus faecalis* | Amp^S^, benzylpenicillin^S^, vancomycin^S^, ciprofloxacin^S^, erythromycin^I^, Gm^S^, levofloxacin^I^, linezolid^S^, nitrofurantoin^S^, **quinurpristin/dalfopristin^R^**, streptomycin^S^, **tetracycline^R^**, tigecycline^S^ |
| *Klebsiella oxytoca* | **ESBL+**, Amikacin^S^, **Amp^R^, Amp/sulbactam^R^**, **cefazolin^R^**, **cefepime^R^**, **cefoxitin^R^**, **ceftazidime^R^**, **ceftriaxone^R^, ciprofloxacin^R^**, ertapenem^S^, Gm^I^, imipenem^S^, **levofloxacin^R^**, nitrofurantoin^I^, **piperacillin/tazobactam^R^**, tobramycin^I^, **trimethoprim/sulfamethoxazole^R^** |
| *Serratia marcescens* | Amikacin^S^, **Amp^R^, Amp/sulbactam^R^**, **cefazolin^R^**, **cefepime^R^**, **cefoxitin^R^**, **ceftazidime^R^**, **ceftriaxone^R^,** ciprofloxacin^I^, ertapenem^I^, Gm^I^, **imipenem^R^**, levofloxacin^I^, **nitrofurantoin^R^**, **piperacillin/tazobactam^R^**, tobramycin^S^, trimethoprim/sulfamethoxazole^S^ |

**Supplementary Table 2. Clinical scoring system**

| **Score** | **Symptoms** |
| --- | --- |
| 0 | an ambulatory and active, normal fur coat, normal amount of feces and urine |
| 1 | slightly ruffled fur, less active, normal number of fecal pellets and less urine present |
| 2 | ruffled fur, not very active (but moves around), less feces present, little urine |
| 3 | hunched posture, increased respirations, diarrhea, inactive but moves when stimulated, little urine |
| 4 | animal on side, not moving when stimulated, rapid shallow respirations |


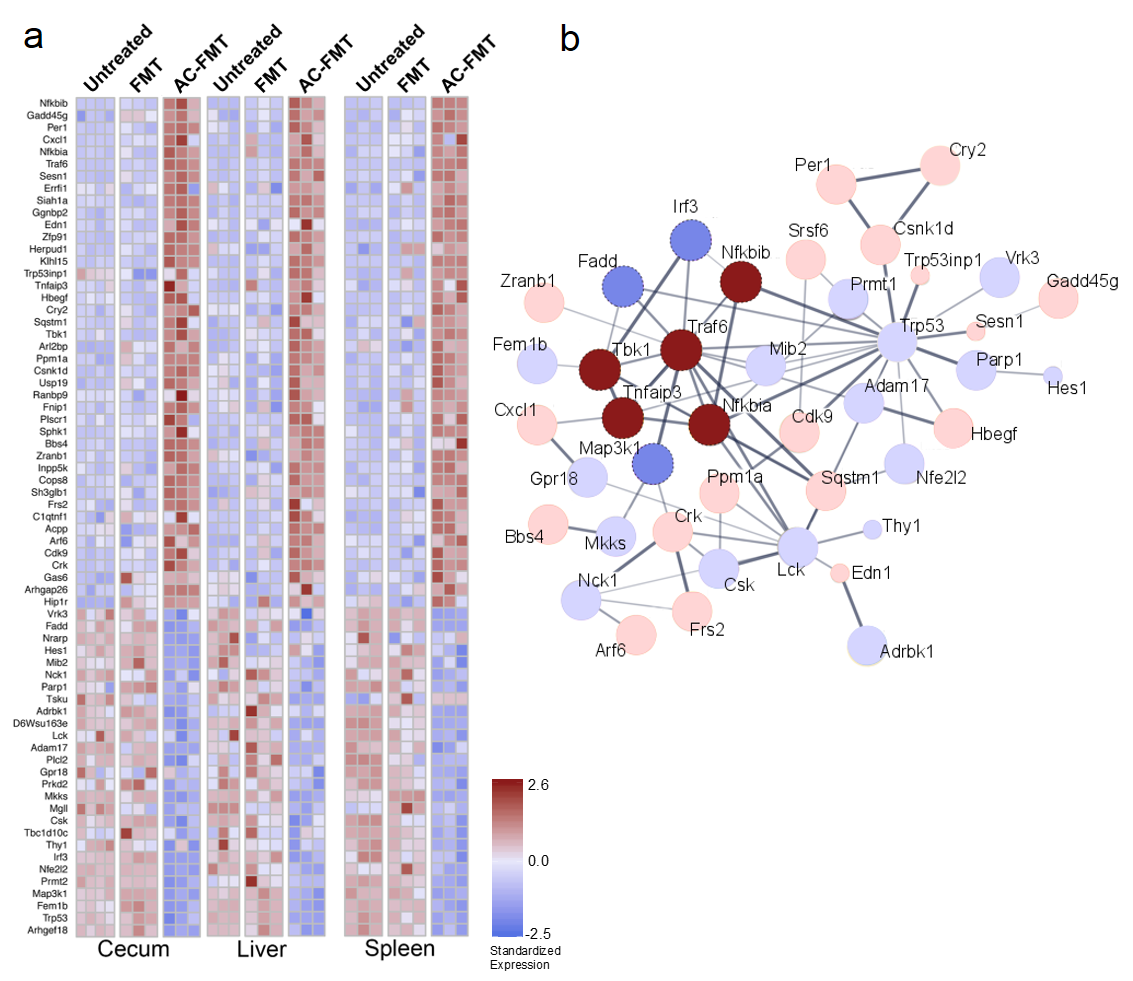


**Supplementary Figure 1. FMT reverses the dysregulated host transcriptional response to a four-member pathogen community isolated from a patient with lethal sepsis.** (**a,b**) The cecum, liver, and spleen of Surgery + PC + FMT or Surgery + PC + AC-FMT treated mice was harvested on POD2, mRNA was extracted from whole tissues, and gene expression was measured using whole genome microarray and qPCR. Gene expression was compared against the baseline of untreated control mice (denoted ‘Untreated’; n = 3 mice / group). In order to define the universal host response to sepsis while dissociating organ-specific effects, we focused on genes that were coordinately regulated across organs. We used stringent cutoffs to identify a set of 299 genes that showed a significant response in AC-FMT (FDR < 0.01 and |log2 fold change| > 0.5) but *not* in FMT treated mice (FDR > 0.5; **Supplementary Data 1**). These genes were significantly enriched among genes involved in the regulation of intracellular signal transduction (FDR = 6.3x10^-4^), several metabolic processes (FDR ≤ 0.01), and the regulation of response to stimulus (FDR = 4.1x10^-2^) (**Supplementary Data 2**). We next used the STRING database to look for known functional interactions among genes involved in the response to stimulus (GO term 0048583) that are changed uniquely in AC-FMT treated mice progressing towards severe sepsis and death (**a)**. Among the 69 genes tested, 42 belong to a tightly connected functional network that contains several master regulators of inflammation (**b**). Analysis of this network revealed an unexpected pattern suggesting immune subversion by the PC that was prevented by FMT. *Transformation related protein 53 (Trp53)*, a central node in the functional network that interacts with 17 other proteins (**b**) was downregulated in AC-FMT but not FMT treated mice, supporting previous reports that bacterial pathogens subvert the Trp53 pathway to promote their own growth. Furthermore, we observed a concomitant blockade of the *Nuclear Factor Kappa B (NF-kB)* and *Interferon Regulatory Factor 3* (IRF3) pathways (**a,b**), two central signaling pathways involved in protective immunity against pathogens, downstream of toll-like receptors and other pathogen recognition receptors (PRRs)^18,19,22,23,24^. More specifically, *NF-kB Inhibitor Alpha* (*NFKBIA)*, *NF-kB Inhibitor Beta (NFKBIB), and TNF Alpha Induced Protein 3* *(TNFAIP3)*, known inhibitors of NF-kB signaling^23,24^, were upregulated (**a,b**). In addition, and indicative of the overall control of host-protective immune pathways by PC, *Mitogen-Activated Protein Kinase Kinase Kinase 1 (Map3k1)* was also downregulated (**a,b**). In agreement with an increased bacterial burden in AC-FMT treated mice, *TANK Binding Kinase 1 (TBK1)* and *TNF Receptor Associated Factor 6 (TRAF6)*, which operate downstream of Toll-like receptors and upstream of NF-kB and IRF3, were upregulated (**a,b**).


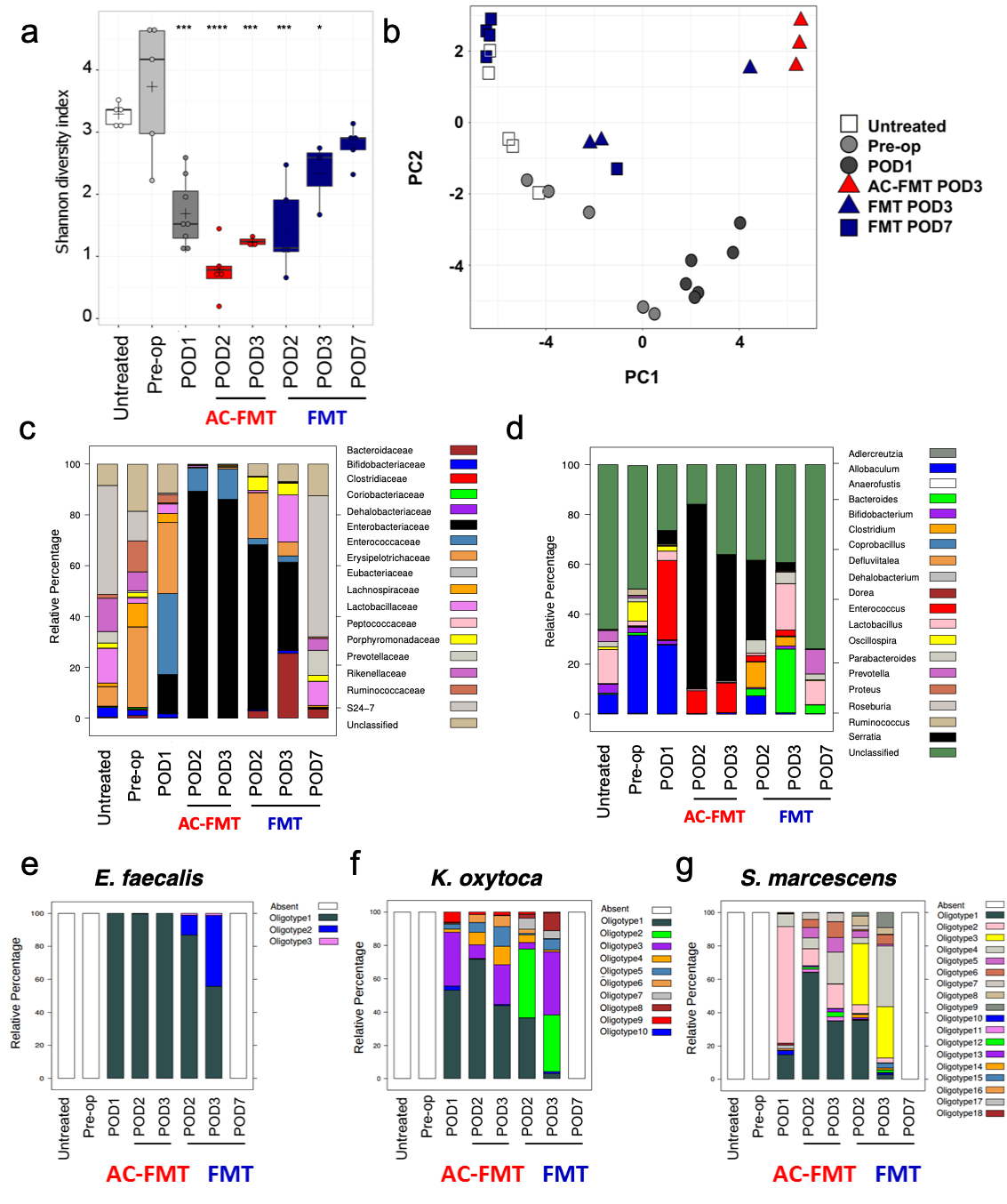


**Supplementary Figure 2. FMT drives the restoration of microbial diversity and the clearance of pathogens in the gut.** (**a-g**) To investigate the impact of FMT on the gut microbiota of mice with gut derived sepsis, 16S rRNA sequencing was performed on cecal contents in a temporal manner, on post-operative day (POD) 1, 2, 3, and 7 in mice that were treated with Surgery + PC + FMT or Surgery + PC + AC-FMT. ‘Preop’ are control mice that were starved and injected intramuscularly with antibiotics but not given surgery or PC (n = 5, 5, 8, 5, 3, 5, 3, and 5 for Untreated, Pre-op, POD1, AC-FMT POD2, AC-FMT POD3, FMT POD2, FMT POD3, and FMT POD7 mice, respectively).

(**a**) Boxplots of alpha diversity (Shannon) of bacterial community diversity for the treatment groups indicated (center line is median, plus symbol is mean, box limits are upper and lower quartiles, whiskers at 1.5x interquartile range; points Pairwise ANOVA vs Untreated group indicated above boxplots; *P ≤ 0.05, ***P ≤ 0.001; ****P ≤ 0.0001; all pairwise comparisons detailed in Supplementary Table 3).

(**b**) Principal coordinates analysis (PCoA) based on the weighted UniFrac distance between the bacterial communities from the treatment groups indicated (PERMANOVA, P = 0.027, R^2^ = 0.415).

(**c**) Bacterial community composition is shown by relative abundance of taxa at Family level.

(**d**) Bacterial community composition is shown by relative abundance of taxa at Genus level.

(**e-f**) Oligotyping analysis^45^ (methodology clarified in Materials and Methods) of *S. marcescens* (**e**), *E. faecalis* (**f**), and *K. oxytoca* (**g**). Dark grey color columns representing oligotype 1 for each of these species with 100% similarity to oligotypes of community strains derived based on the whole genome sequence data.

**Supplementary Table 3. Pairwise ANOVA for 16S rRNA alpha diversity in gut derived sepsis model**

| **measure** | **From** | **to** | **y** | **p** |
| --- | --- | --- | --- | --- |
| Simpson | POD1 | AC-FMT POD2 | 1.063550777 | 0.0032 |
| Simpson | POD1 | FMT POD7 | 1.160237212 | 0.00032 |
| Simpson | POD1 | Untreated | 1.256923646 | 3.40E-05 |
| Simpson | POD1 | FMT POD3 | 1.35361008 | 0.0033 |
| Simpson | POD1 | FMT POD2 | 1.450296515 | 0.0027 |
| Simpson | POD1 | AC-FMT POD3 | 1.546982949 | 0.0023 |
| Simpson | AC-FMT POD2 | Untreated | 1.643669383 | 0.0011 |
| Simpson | Untreated | FMT POD3 | 1.740355818 | 0.046 |
| Simpson | Untreated | FMT POD2 | 1.837042252 | 0.016 |
| Simpson | Untreated | AC-FMT POD3 | 1.933728686 | 0.00023 |
| Shannon | POD1 | FMT POD7 | 5.00790357 | 0.0066 |
| Shannon | POD1 | Untreated | 5.46316753 | 0.00025 |
| Shannon | POD1 | FMT POD3 | 5.918431491 | 0.036 |
| Shannon | AC-FMT POD2 | FMT POD7 | 6.373695452 | 0.0036 |
| Shannon | AC-FMT POD2 | Untreated | 6.828959413 | 2.70E-05 |
| Shannon | AC-FMT POD2 | FMT POD3 | 7.284223374 | 0.016 |
| Shannon | FMT POD7 | Untreated | 7.739487335 | 0.013 |
| Shannon | FMT POD7 | FMT POD2 | 8.194751296 | 0.033 |
| Shannon | Untreated | FMT POD3 | 8.650015256 | 0.014 |
| Shannon | Untreated | FMT POD2 | 9.105279217 | 0.00037 |
| Shannon | Untreated | AC-FMT POD3 | 9.560543178 | 0.00019 |


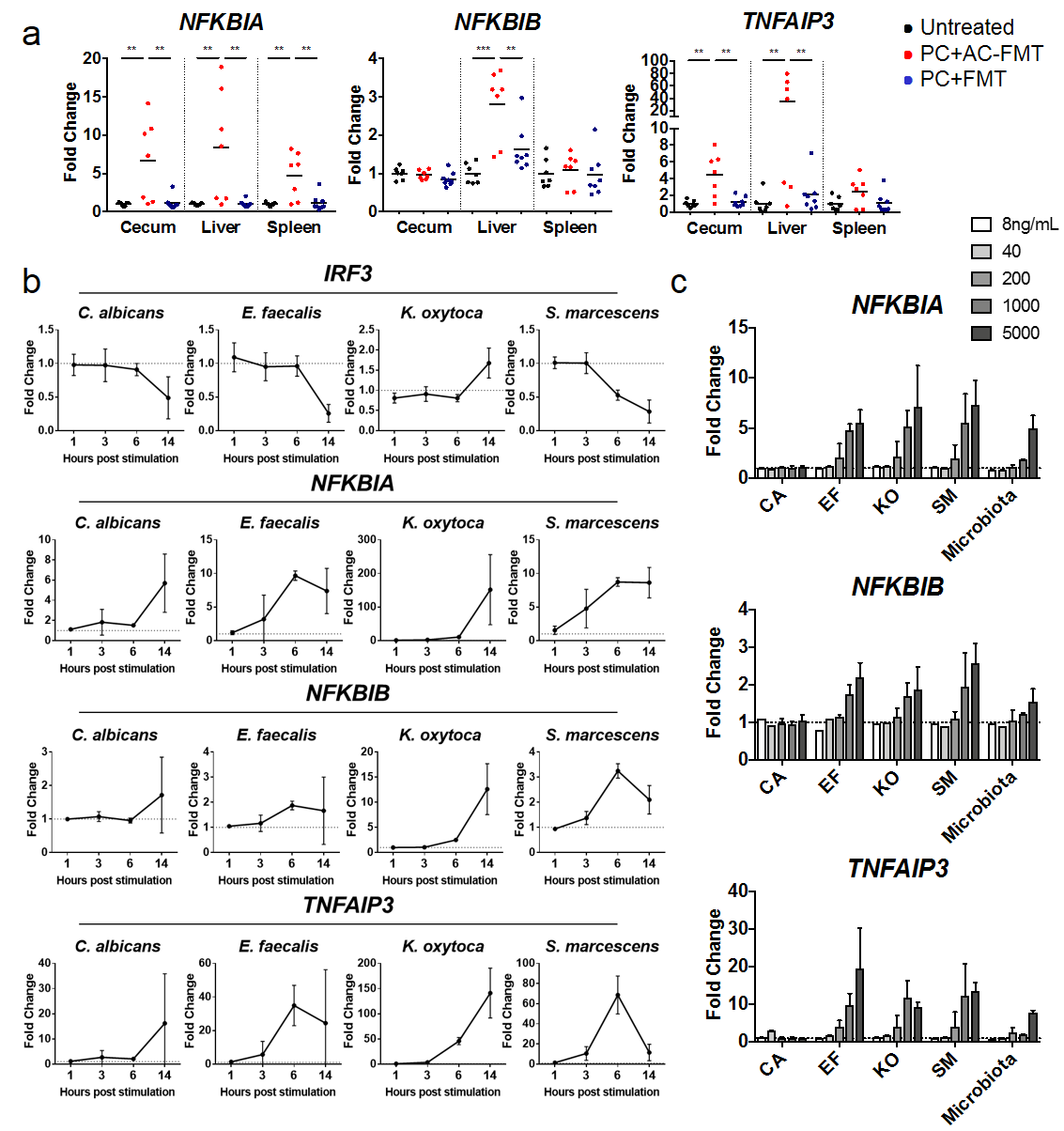


**Supplementary Figure 3. The pathogen community causes the dysregulation of innate immunity.** (**a**) Mice were injected IP with PC and treated with FMT or AC-FMT as before. qPCR for *NFKBIA, NFKBIB* and *TNFAIP3* on RNA isolated from indicated organs approximately 20 hours post injection of PC (Fold change compared to the mean of untreated group relative expression for each gene and organ; n = 7, 7, and 8 for Untreated, AC-FMT, and FMT treated mice, respectively; center is mean; One-way ANOVA / Tukey’s multiple comparison; **P ≤ 0.01, ***P ≤ 0.001).

(**b**) MEFs were cultured for 1, 3, 6 and 14 hours in culture media containing individual PC members. RNA was isolated and qPCR was performed to measure the effect of PC members on *IRF3*, *NFKBIA, NFKBIB,* and *TNFAIP3* expression *in vitro*. Gene expression in response to PC co-culture is displayed as fold change compared to gene expression of untreated MEFs harvested at the same time point (e.g. *IRF3* expression of MEFs cultured with C. albicans for 14 hours compared to IRF3 expression of untreated MEFs cultured for 14 hours) (3 independent experiments; n ≥ 3 for all timepoints; mean ± SD).

(**c**) MEFs were cultured for 12 hours in culture media containing filtered lysates made individual PC members, or from cecal contents of Untreated mice at the indicated concentrations. MEF RNA was isolated and qPCR was performed to measure the effect of PC members on *NFKBIA, NFKBIB* and *TNFAIP3* expression *in vitro*. (Fold change compared to the baseline of untreated MEF *NFKBIA, NFKBIB,* and *TNFAIP3* relative expression [indicated with the dotted line at y = 1] shown; 3 independent experiments; n ≥ 3 for all conditions; mean + SD).

**Supplementary Table 4. qPCR primer sequences**

| **Gene** | **Forward primer (5’-3’)** |
| --- | --- |
|  | **Reverse primer (3’-5’)** |
| *IRF3* | GAGAGCCGAACGAGGTTCAG |
|  | CTTCCAGGTTGACACGTCCG |
| *NFKBIA* | TGAAGGACGAGGAGTACGAGC |
|  | TTCGTGGATGATTGCCAAGTG |
| *NFKBIB* | GCGGATGCCGATGAATGGT |
|  | TGACGTAGCCAAAGACTAAGGG |
| *TNFAIP3* | ACCATGCACCGATACACGC |
|  | AGCCACGAGCTTCCTGACT |
| *GAPDH* | AGGTCGGTGTGAACGGATTTG |
|  | TGTAGACCATGTAGTTGAGGTCA |
| *HPRT* | CCTAAGATGAGCGCAAGTTGA |
|  | CCACAGGACTAGAACACCTGCTAA |
| *ASL* | TCTTCGTTAGCTGGCAACTCACCT |
|  | ATGACCCAGCAGCTAAGCAGATCA |
| Universal 16S | ACTCCTACGGGAGGCAGCAGT |
|  | ATTACCGCGGCTGCTGGC |

**Supplementary Figure 4. IRF3 plays a role in protective immunity.** Mice were injected IP with PC and treated with AC-FMT as before. Kaplan-Meier survival curves (n = 7 for IRF3^+/+^ AC-FMT, 6 for IRF3^+/-^ AC-FMT, 11 for IRF3^-/-^ AC-FMT; Log-rank (Mantel-Cox) test, P = 0.4897 between IRF3^+/+^ AC-FMT and IRF3^+/-^ AC-FMT, P = 0.1269 between IRF3^+/+^ AC-FMT and IRF3^-/-^ AC-FMT, P = 0.7186 between IRF3^+/-^ AC-FMT and IRF3^-/-^ AC-FMT).

**Supplementary Figure 5. The microbiome composition of mice with systemically disseminated pathogens is largely unperturbed.** (**a**-**c**) Mice were injected IP with PC and treated with FMT or AC-FMT as before. Cecal contents were collected approximately 20 hours post injection of PC.

(**a**) Bacterial DNA was extracted, and bacterial load was assessed by performing qPCR on 16S copies (universal 16S primers) normalized to host housekeeping gene ASL (n = 16, 25, 8, and 10 for Untreated, PC, PC + AC-FMT, and PC + FMT treated mice, respectively; center is mean; One-way ANOVA / Tukey’s multiple comparison; p > 0.05 between all groups).

(**b**) 16S rRNA sequencing was performed and boxplots of alpha diversity (Shannon) of bacterial community diversity for the treatment groups indicated (n = 5 mice / group; center line is median, plus symbol is mean, box limits are upper and lower quartiles, whiskers at 1.5x interquartile range; no significant different differences seen by pairwise ANOVA).

(**c**) 16S rRNA sequencing was performed and bacterial community composition is shown by relative abundance of taxa at Genus level (n = 5 mice / group). The AC-FMT (inoc.) and FMT (inoc.) represent 16S rRNA sequencing of the inoculum that was used in this experiment to treat the mice from the PC+AC-FMT and PC+FMT groups.

**Supplementary Figure 6. SCFA levels and butyrate producing OTUs in the feces of pathogen infected mice.** (**a,b**) Mice were injected IP with PC and treated with FMT or AC-FMT as before. Cecal contents were collected approximately 20 hours post injection of PC. (**a**) Acetate and (**b**) propionate levels were measured by GC-MS (n = 9, 10, 8, and 10 for Untreated, PC, PC + AC-FMT, and PC + FMT treated mice, respectively; One-way ANOVA / Tukey’s multiple comparison; *P ≤ 0.05, **P ≤ 0.01).

(**c,d**) Analysis of the OTUs found in the datasets reveals that FMT increases the presence and abundance of butyrate-producing bacteria. Changes in specific OTUs between mice treated with or without FMT were detected using the R packages phyloseq and DESeq2^50,51^. The figure depicts significant log2-fold changes based on an FDR (Benjamini–Hochberg) significance threshold of 0.01 in the relative abundance of specific OTUs between pairwise comparisons of Untreated, PC, and PC+FMT mice as heatmaps. (c) Untreated versus PC mice and (d) Untreated versus PC+FMT mice were compared. Representative sequences for each of these OTUs were then used to search the Ribosomal Database Project’s set of quality-controlled, aligned and annotated bacterial 16S rRNA sequences using SeqMatch^36^ in order to obtain nearest neighbor matches with cultured representatives at the genus-species level. On the left are the phylum and family classifications of the OTUs and on the right are the putative genus/species designation (numbers in parentheses indicate SeqMatch score [Sab]). The panel to the right of the heatmaps indicate whether butyrate kinase (buk) or butyryl-CoA:acetate CoA-transferase (but) encoding genes have been detected in the genomes of the SeqMatch genus/species assignments based on the *buk* and *but* entries in the RDP FunGene Functional Gene Pipeline and Repository^52^. A closed black circle indicates presence of a buk or but gene in that genome, whereas a closed gray circle indicates an alternate annotation (defined at the bottom of the figure). An open circle indicates that no match was found in the RDP FunGene. Those listed in bold on the right are enriched in the PC+FMT mice.
