# Supplementary material for: Fecal microbiota transplant rescues mice from human pathogen mediated sepsis by restoring systemic immunity
Source: Nat Commun. 2020 May 11;11:2354. doi: 10.1038/s41467-020-15545-w (PMC7214422; doi:10.1038/s41467-020-15545-w)
Supplement: Supplementary file 3 — Description of Additional Supplementary Files [file 41467_2020_15545_MOESM3_ESM.docx]

**Description of Additional Supplementary Files**

File Name: Supplementary Data 1

Description: List of differently expressed genes in response AC-FMT and FMT across all tissues and within each of the tissues studied

File Name: Supplementary Data 2

Description: Gene set enrichment analysis for genes showing a significant response in AC-FMT but not in FMT treated mice as compared to untreated animals

File Name: Supplementary Data 3

Description: Analysis of the OTUs found in the datasets reveals that FMT increases the presence and abundance of butyrate-producing bacteria
