# Supplementary material for: Fecal microbiota transplant rescues mice from human pathogen mediated sepsis by restoring systemic immunity
Source: Nat Commun. 2020 May 11;11:2354. doi: 10.1038/s41467-020-15545-w (PMC7214422; doi:10.1038/s41467-020-15545-w)
Supplement: Supplementary file 7 — Reporting Summary [file 41467_2020_15545_MOESM7_ESM.pdf]

nature research

Corresponding author(s): John C. Alverdy and Bana Jabri

Reporting Summary

Nature Research wishes to improve the reproducibility of the work that we publish. This form provides structure for consistency and transparency in reporting. For further information on Nature Research policies, see Authors & Referees and the Editorial Policy Checklist.

Statistical parameters

When statistical analyses are reported, confirm that the following items are present in the relevant location (e.g. figure legend, table legend, main text, or Methods section).

n/a	Confirmed	
☐	☒	The exact sample size (*n*) for each experimental group/condition, given as a discrete number and unit of measurement
☐	☒	An indication of whether measurements were taken from distinct samples or whether the same sample was measured repeatedly
☐	☒	The statistical test(s) used AND whether they are one- or two-sided<br>*Only common tests should be described solely by name; describe more complex techniques in the Methods section.*
☐	☒	A description of all covariates tested
☐	☒	A description of any assumptions or corrections, such as tests of normality and adjustment for multiple comparisons
☐	☒	A full description of the statistics including central tendency (e.g. means) or other basic estimates (e.g. regression coefficient) AND variation (e.g. standard deviation) or associated estimates of uncertainty (e.g. confidence intervals)
☐	☒	For null hypothesis testing, the test statistic (e.g. *F*, *t*, *r*) with confidence intervals, effect sizes, degrees of freedom and *P* value noted<br>*Give P values as exact values whenever suitable.*
☒	☐	For Bayesian analysis, information on the choice of priors and Markov chain Monte Carlo settings
☒	☐	For hierarchical and complex designs, identification of the appropriate level for tests and full reporting of outcomes
☒	☐	Estimates of effect sizes (e.g. Cohen's *d*, Pearson's *r*), indicating how they were calculated
☐	☒	Clearly defined error bars<br>*State explicitly what error bars represent (e.g. SD, SE, CI)*

Our web collection on statistics for biologists may be useful.

Software and code

Policy information about availability of computer code

Data collection	For quantitative PCR, LightCycler® 480 Software was used.
Data analysis	Gene transcriptional analysis was performed in R, using the Bioconductor software package lumi, the Bioconductor limma package, and Gene Ontology (GO) enrichment analysis were done using Gorilla. 16S rRNA analysis was performed using the UPARSE pipeline, the "parallel_assign_taxonomy_rdp.py" script of QIIME software, PyNast and FastTree, and the oligotyping pipeline. GraphPad Prism, Excel were used for all other analysis (see Methods for additional details).

For manuscripts utilizing custom algorithms or software that are central to the research but not yet described in published literature, software must be made available to editors/reviewers upon request. We strongly encourage code deposition in a community repository (e.g. GitHub). See the Nature Research guidelines for submitting code & software for further information.

Data

Policy information about availability of data

All manuscripts must include a data availability statement. This statement should provide the following information, where applicable:

- Accession codes, unique identifiers, or web links for publicly available datasets
- A list of figures that have associated raw data
- A description of any restrictions on data availability

The datasets generated during and/or analyzed during the current study are available in the Article, Supplementary Information files or available from the corresponding author on reasonable request. Microarray data was uploaded to the NCBI Gene Expression Omnibus database, GEO accession number: GSE71530 [https://www.ncbi.nlm.nih.gov/geo/query/acc.cgi?acc=GSE71530]. Genome sequences of the ICU1-2 pathogen community members were uploaded to NCBI in BioProject PRJNA307050 [https://www.ncbi.nlm.nih.gov/bioproject/?term=PRJNA307050].

Field-specific reporting

Please select the best fit for your research. If you are not sure, read the appropriate sections before making your selection.

☒ Life sciences ☐ Behavioural & social sciences ☐ Ecological, evolutionary & environmental sciences

For a reference copy of the document with all sections, see nature.com/authors/policies/ReportingSummary-flat.pdf

Life sciences study design

All studies must disclose on these points even when the disclosure is negative.

Sample size	The majority of experiments were repeated at least three times to obtain data for indicated statistical analyses. Mice were allocated to experimental groups on the basis of their genotype and randomized within the given age-matched group. Given that our mice were inbred and matched for age, we always assumed similar variance between the different experimental groups. We did not perform an a priori sample size estimation but always used as many mice per group as possible in an attempt to minimize type I and type II errors.
Data exclusions	All experimental and control animals were littermates and none were excluded from the analysis at the time of harvest.
Replication	The majority of experiments were repeated at least three times to ensure reproducibility. Statistical analyses were done to illustrate significance. All attempts to replicate experiments were successful.
Randomization	Mice were allocated to experimental groups on the basis of their genotype and randomized within the given age-matched group.
Blinding	Investigators were not blinded during experiments and outcome assessment.

Reporting for specific materials, systems and methods

Materials & experimental systems

n/a	Involved in the study	
☒	☐	Unique biological materials
☒	☐	Antibodies
☐	☒	Eukaryotic cell lines
☒	☐	Palaeontology
☐	☒	Animals and other organisms
☒	☐	Human research participants

Methods

n/a	Involved in the study	
☒	☐	ChIP-seq
☒	☐	Flow cytometry
☒	☐	MRI-based neuroimaging

Eukaryotic cell lines

Policy information about cell lines

Cell line source(s)	Mouse embryonic fibroblasts
Authentication	Self generated - no separated authentication was performed.
Mycoplasma contamination	All cell lines tested negative for mycoplasma (tested using HEK-Blue™ TLR2 cells from Invivogen).
Commonly misidentified lines (See ICLAC register)	*Name any commonly misidentified cell lines used in the study and provide a rationale for their use.*

Animals and other organisms

Policy information about studies involving animals; ARRIVE guidelines recommended for reporting animal research

Laboratory animals	Seven to nine-week old male C57BL/6 mice weighing 18-22 g were used for experiments. WT C57BL/6 were purchased from Charles River Laboratories. IRF3-/- mice on a C57BL/6 background were kindly provided by Dr. Tatyana V. Golovkina (University of Chicago) and mice used in comparative studies were the progeny of IRF3+/- mice resulting in IRF3+/+ and IRF3-/- littermates. IRF3+/+ and IRF3-/- littermates were cohoused until the day of the experiment to prevent microbiome related differences between genotypes from affecting results.
Wild animals	This study did not involve any wild animals.
Field-collected samples	This study did not involve any field-collected samples.
